# Alzheimer’s Disease: A Molecular Model and Implied Path to Improved Therapy

**DOI:** 10.3390/ijms25063479

**Published:** 2024-03-20

**Authors:** Meagan Susanne Weaver-Rosen, Philip Serwer

**Affiliations:** 1 Department of Microbiology, Immunology and Molecular Genetics, UT Health, San Antonio, TX 78229, USA; weaverrosen@livemail.uthscsa.edu; 2 Department of Biochemistry and Structural Biology, UT Health, San Antonio, TX 78229, USA

**Keywords:** agarose gel electrophoresis, native, amyloid-forming proteins, disease modeling, drug effectiveness assay, molecular model, alpha-sheet

## Abstract

Amyloid-associated neurodegenerative diseases, including Alzheimer’s disease (AD), are characterized by the in-brain accumulation of β-sheet structured protein aggregates called amyloids. However, neither a disease model nor therapy is established. We review past data and present new, preliminary data and opinions to help solve this problem. The following is the data-derived model/hypothesis. (1) Amyloid-forming proteins have innate immunity functions implemented by conversion to another sheet conformation, α-sheet. (2) In health, α-sheet structured, amyloid-forming proteins inactivate microbes by co-assembly with microbe α-sheets. Amyloid-forming proteins then undergo α-to-β-sheet conversion. (3) In disease, α-sheet-structured, amyloid-forming proteins over-accumulate and are neuron-toxic. This hypothesis includes formation by virus capsid subunits of α-sheets. In support, we find that 5–10 mM methylene blue (MB) at 54 °C has a hyper-expanding, thinning effect on the phage T4 capsid, as seen by negative stain- and cryo-electron microscopy after initial detection by native gel electrophoresis (AGE). Given the reported mild anti-AD effect of MB, we propose the following corollary hypothesis. (1) Anti-AD MB activity is, at least in part, caused by MB-binding to amyloid α-sheet and (2) MB induces the transition to α-sheet of T4 capsid subunits. We propose using AGE of drug incubated T4 to test for improved anti-AD activity.

## 1. Introduction: Previous Data and Our Best-Fit Model

### 1.1. Protein Conformation and Alzheimer’s Disease

Several neurodegenerative diseases, including Alzheimer’s disease (AD), are usually associated with excess in-brain production of extracellular, β-sheet-structured proteins, usually thought to be causative. This protein is generically called amyloid because of its starch-like texture (recently reviewed [1,2,3,4,5]). Therapies for AD are usually designed to promote the removal or destruction of amyloid proteins (Aβ protein in the case of AD) or their cleavage products, based on the hypothesis that the observed amyloid causes disease (amyloid cascade hypothesis [6,7,8]). However, these therapies have failed to significantly reverse the course of AD [7,8,9,10,11,12,13].

Some evidence implies that β-sheet-structured amyloid proteins are not in the pathway of disease formation. For example, AD-related symptoms sometimes occur without excess β-sheet amyloid protein production [14,15,16], and excess β-sheet amyloid (plaque) production sometimes occurs without AD [17,18] (review [12]). Possibly, β-sheet-structured amyloid proteins are a dead-end product of protein conformation-based detoxification [19]. In other words, AD is a protein conformation- and amount-caused disease, as, by inference, would be other amyloid-associated neurodegenerative diseases.

Given the above scenario, electron microscopic observation of AD brain might be revealing. The perspective involved has some similarity to the perspective that generated the following quotation attributed [20] to physicist Richard Feynman: “It is very easy to answer many of these fundamental biological questions; you just look at the thing!” One observation, made by electron microscopy of thin sections, is that at least some dark lipofuscin of an AD brain is packed with bent, doubled fibers [21]. In contrast, non-AD brain has dark lipofuscin in which bent, doubled fibers appear to be relatively rare [22]. Thus, the bent, doubled fibers are candidates for being the toxic agent in AD. More extensive statistics are needed in this (underdeveloped) area.

The proposal had previously been made (recently [23,24,25]) that AD is caused by a toxic protein conformation called α-sheet, a protein conformation discovered by Pauling and Corey [26,27] before they discovered the well-known β-sheet. Molecular dynamics simulation has shown that α-sheets and β-sheets can interconvert [25,28,29], with interconversion stimulated by side-chain hydrophilicity [25]. The α-sheet-as-disease-causing-agent proposal is dramatically supported by the observation that the toxic form of in vitro-assembled AD amyloid proteins (cleaved Aβ protein, 40–42 amino acids long; review [28,29]) have two characteristics of α-sheets: (1) circular dichroism near zero and (2) an appropriate NMR signature [23,24,25]. Importantly, molecular dynamics simulation reveals that single polypeptide chains in α-sheet conformation are bent to avoid steric side chain clashes [28], a situation anticipated by Pauling and Cory [26,27]. The existence of the steric clashes is the probable reason that α-sheets are relatively rare and unknown. A reasonable opinion is that further exploration should be made of a possible linkage of AD to the bent fibers seen in the AD brain by electron microscopy.

A second possible reason for the obscurity of α-sheets is that the empirical detection of α-sheets has occurred only for 4–6 amino acid-long stretches, for example, in ATP-binding sites, metal binding sites and phosphate binding sites. In these cases, the α-sheet is bent [30,31]. Longer α-sheets have been seen only by molecular dynamics simulation (recent review [24,25]). Pauling and Corey proposed α-sheet bending to be the reason that bird feathers are bent, although they did not use the term α-sheet [27]. The absence of long (>6 amino acid) stretches of α-sheets in human proteins makes long α-sheets a potential innate immunity target in that targeting long α-sheets will not damage healthy functions. 

Inter-conversion of α- and β-sheet polypeptides occurs by rotations around C–N and C–C bonds that generate an approximately 180° rotation of every other amino acid of a polypeptide projected to two dimensions [25,26,28]. Thus, in addition to bending, a second α-sheet characteristic is the presence of all α-carboxyl groups on one edge and the presence of all α-amino groups on the opposing edge of a polypeptide chain. At physiological pH, this implies a net separation of negative and positive charges along the surface of the polypeptide. In contrast, along the surface of a β-sheet, α-carboxyl and α-amino groups alternate so that this charge separation does not occur [25,26,28].

### 1.2. Viruses, α-Sheets and Alzheimer’s Disease

Independently, a previous study [32] revealed a phage T3 capsid with incompletely packaged DNA and an outer protein shell that had undergone hyper-expansion. The hyper-expansion implied thinning of the outer shell, which strongly suggested either β-sheet or α-sheet structures of the subunits of the shell [32,33]. However, this capsid also had an unusually high negative charge on its surface, suggesting the presence of the charge separation characteristic of α-sheets [33]. Of course, the surfaces of viral capsids are also bent, which fits well with α-sheet structured subunits. The assumption of an α-sheet structure also provides a possible mechanism for the size dynamics of the capsid (Figure 2 of [33]).

Furthermore, electron microscopy of thin sections has revealed that these hyper-expanded capsids are enhanced in T3-infected cells by (1) agarose gel-embedding during infection and (2) the addition of proflavine to the agarose-embedded, infected cells. Proflavine is a dye known to inhibit DNA packaging. Thus, the hyper-expanded capsids are thought to be products of DNA packaging, and possibly are an intermediate of a backup DNA packaging motor [34].

The proposal has been made [19,21] that these observations are linked to the observations of others [35,36,37,38,39,40] that (1) herpesvirus infections are, statistically speaking, precursors of AD, and (2) this precursor–product relationship is dependent on the presence of the ApoE-ε4 allele. (This context dependence implies that the procedure used was capable of detecting no herpesvirus dependence when it existed.) The proposed linkage of these two observations is via (1) the sequence homology and structural homology-based conclusion that phages and herpesviruses have the same DNA packaging pathway (review [34]) and (2) the effects of the ApoE-ε4 allele on the composition of gel-promoting proteins in the basement membrane, a gel that surrounds neuronal cells [41]. The hypothesis is that the α-sheet-generating effect of gel-embedding of phage T3-infected bacteria is mimicked (details not proposed) by alteration of the basement membrane around cells in which herpesviruses are propagating.

The studies of the virus infection association of AD do not, to our knowledge, consider either of the following hypothetical possibilities. (1) A virus assembly-associated version of the viral capsid (not the virus) is the AD trigger. (2) The triggering of symptoms is protein conformation-based, specifically, α-sheet-based. In our opinion, the integration of data of various types is the key step needed to form a more complete understanding of the disease and possible therapies.

### 1.3. Integration of Data to Develop a Disease Model

Based on three sources of key evidence, in this section, we integrate the above data to generate a molecular pathway for both AD and other amyloid-associated, neurodegenerative diseases. The first source of key evidence is the observation that amyloid proteins are related to viral proteins [42]. This observation supports the proposal that amyloid-associated, neurodegenerative diseases are caused by activities of an amyloid protein-dependent innate immune system. The proposed [19] origins of this system are previous virus infections, with selection for the retention of amyloid proteins via the proteins’ countering of future virus infections. CRISPR [43,44] is analogous at the level of DNA.

The second source of key evidence is a collection of data that further support the previously detected dependence of some AD on previous virus infection, especially with herpesviruses. These data indicate that (discussion of details in [19]), since ~1995, age-normalized AD frequency (not AD frequency without age normalization) has decreased in the United States and Western Europe but not in China and Japan [45], where anti-herpes virus vaccination has been less emphasized. These results and others [35,46,47] demonstrate a correlation of lower AD incidence with the introduction of anti-herpesvirus (and other anti-virus [46,47]) measures.

The third source of key evidence is the collection of observations, discussed above, that indicate (1) similarity of phage DNA packaging to herpesvirus DNA packaging and (2) in-gel propagation-enhanced, DNA packaging-associated phage T3 capsid hyper-expansion, which is likely to include the conversion of capsid subunits to α-sheet conformation. Thus, a reasonable proposal is that herpesviruses (1) assemble the DNA packaging-associated particles that phages assemble, including the hyper-expanded capsid, for which α-sheet-structured subunits are proposed; (2) stimulate the anti-viral, innate immune conversion to α-sheet structure of amyloid proteins; and (3) are, via this mechanism, triggers of neurodegenerative diseases, including AD.

**Figure 1:**
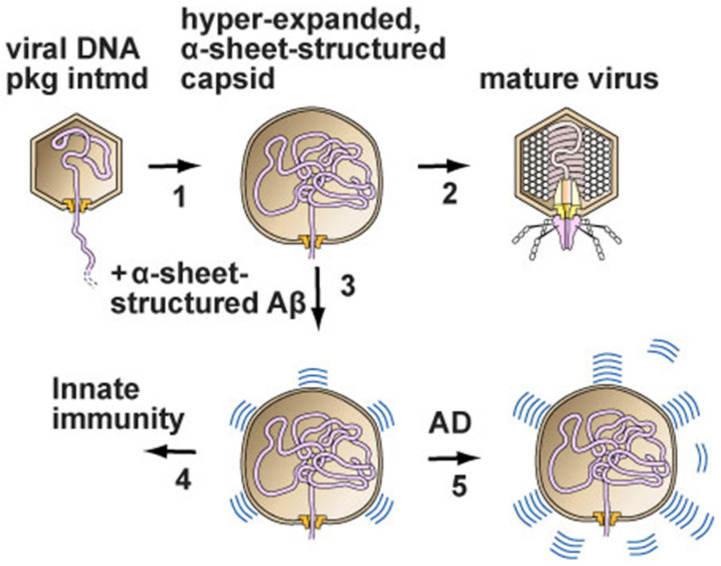
The disease model deduced from diverse data, as described here. (**1**) DNA enters the capsid of either a phage (phage T3 is used as an example) or a herpesvirus. Hyper-expansion, with subunit conversion to an α-sheet structure, occurs to accelerate DNA packaging when packaging is slowed (proposed details in [19,32]). (**2**) In the case of a phage, packaging finishes and a tail is added to the capsid to form a mature phage particle. (**3**,**4**) In the case of herpesviruses, amyloid proteins block the progression of the hyper-expanded capsids by converting to an α-sheet structure and then co-assembling with the α-sheet of subunits of the capsid. (**5**) Alzheimer’s disease is initiated by the over-production of amyloid protein α-sheets, which are toxic. Some amyloid protein α-sheets are subsequently converted to the β-sheet amyloid protein of plaques. However, this conversion is not sufficient to avoid toxicity when Alzheimer’s disease is present (image adapted from [19]).

In summary, the proposed pathway, as deduced from the above, is simple. (1) Virus infection stimulates the conversion of amyloid-forming proteins to α-sheet conformation for the evolved purpose of co-assembling with α-sheet-structured, viral assembly intermediates and, thus, inhibiting infection (left pathway at the bottom of Figure 1). (2) Amyloid protein-associated diseases are caused by a loss of control of the amount of α-sheet conversion that occurs, resulting in the excessive and toxic production of α-sheets (right pathway at the bottom of Figure 1). 

We emphasize that this hypothesis (1) does not include the misfolding of amyloid-forming proteins, as other α-sheet-based hypotheses do (e.g., [24]), and (2) includes an anti-viral function for conversion to α-sheets, as, to our knowledge, first suggested in ref. [42] without structural details, such as α-sheet. Also, the hypothesis of Figure 1 provides the following explanations for hereditary ataxias caused by the over-repeating, sometimes by <10%, of a normally repeated amino acid sequence within a protein that also has a non-repeated amino acid sequence. (1) The reason that these repeats are primarily poly G and poly Q is the formation of α-sheets. Both poly G and poly Q have elevated α-sheet-forming potential [48,49]. (2) Innate immune function is the reason for evolutionary selection for the existence of these amino acid repeats. This existence does not have, to our knowledge, a previously published explanation. (3) The conversion to toxicity by a minor increase in length [48,49] occurs because of the length dependence of the stability of α-sheet conformation.

Details for the above conclusions about poly G and poly Q are the following. Poly G is the only poly-amino acid without side chains and, therefore, readily forms α-sheets [30,31]. Poly Q is the poly-amino acid with the most hydrophilic, electrically neutral side chain [50]. Side-chain hydrophilicity has been found to be a factor that encourages the β- to α-sheet conversion of peptides [25]. The electrical charge of some side chains confers hydrophilicity but might disrupt hydrophobic interactions observed by modeling of the formation of α-sheet by poly Q [49].

### 1.4. The Thin Edge of the Anti-AD Pharmaceutical Wedge

The failure of the drug trials discussed in Section 1.1 does not imply that all drug trials have been complete failures. Limited successes are possible. One way to find the “thin edge of the anti-AD drug wedge” is to search among the trials for relevant limited successes. In our case, relevance would be a linkage to the data and model discussed above. The key challenge is converting limited success into the systematic stopping of the disease process. A possible strategy is discussed below.

Our search did not have to go far to find limited success that has the potential to be linked to the above model. Methylene blue (MB) is (1) the oldest synthetic dye, (2) usable in humans up to about 2 mg/kg body weight and (3) a limited AD suppressor in both humans and animals [51,52,53]. The AD suppression is thought to originate primarily via the enhancement of respiration in mitochondria [52], an effect that MB has been shown to have [54]. However, also possible is that MB binds and detoxifies the toxic conformation of amyloid-forming proteins. Nonetheless, we initially regarded this possibility to be remote enough that we did not pursue it.

However, we appeared to have *accidentally* made a relevant observation while testing the gated loading of anti-cancer compounds in bacteriophage (phage) T4. The idea was to use T4, a phage that has been found to be highly persistent in murine blood, as a gated drug delivery vehicle [55]. We had already successfully achieved gated T4 loading of ethidium and bleomycin without any detected disturbance of the phage capsid [55]. However, when we tested the T4 loading of methylene blue, we made the surprising, possibly AD model-related, discoveries described below. In summary, MB induced a transformation of T4 capsid subunits to an apparent, although not proven, α-sheet-related structure. These observations supported the model of Figure 1 and suggested a path for finding anti-AD drugs more effective than MB.

## 2. The Effect of Methylene Blue (MB) on Phage T4

### 2.1. AGE Analysis

The phage T4 gate is opened by raising the temperature to 54–58 °C and closed by lowering the temperature [55]. The loading is in the in interior of the DNA-containing protein shell (head) of T4. The profile of loaded T4 during native agarose gel electrophoresis (AGE) is not changed by the loading of 7.1 mM bleomycin [55], which is also true at 10.7 mM bleomycin (1416 Da). The reasons for the absence of loading-associated change are the following. (1) Migration during AGE is determined only by the surface of a particle [56]. (2) Loading does not change the surface enough to alter migration during AGE. Ethidium (394 Da) remains loaded when the gate is closed by lowering the temperature to 42 °C or lower [55]. Proof of gate closure is the observation that, after AGE at 25 °C, phage T4 does not stain with the dyes typically used to stain nucleic acids, even though the T4 head contains a dsDNA genome. However, a restoration of phage T4 DNA staining occurs after incubating the gel in conditions that cause the expulsion of packaged T4 DNA molecules [55].

**Figure 2:**
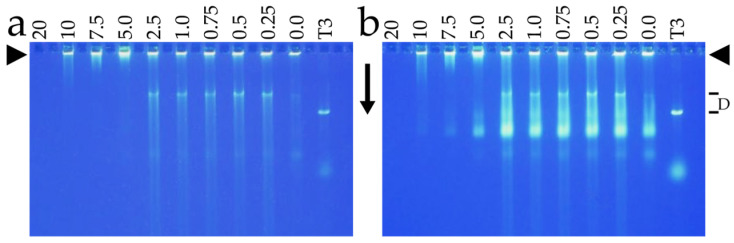
Native agarose gel electrophoresis (AGE) analysis of the interaction of phage T4 with methylene blue (MB). Phage T4 was prepared and partially purified as previously described for the gated loading of ethidium and bleomycin [55]. Then, T4 was incubated with MB at 54 °C for 1.0 h and subjected to horizontal AGE, as described in [55]. (**a**) The gel was photographed after staining with GelStar for 3.0 h [55]. (**b**) The same gel was photographed after the expulsion of packaged DNA by incubation for 18.0 h with 0.002 M EDTA, pH 7.4, at room temperature (22 ± 3 °C). The concentration of MB (mM) is indicated at the top of a lane. The arrowheads indicate the origins of electrophoresis; the arrows indicate the direction of electrophoresis. A phage T3 standard was included (lane labeled T3). Some DNA was spontaneously expelled from both T3 and T4, as indicated by the horizontal lines labeled D at the right.

The following was observed after attempts to load phage T4 with MB and subsequent analysis by (1) AGE/GelStar staining (Figure 2a; MB concentration [mM] is above a lane) and then by (2) GelStar staining of the same gel after expulsion of DNA from phage capsids (Figure 2b). A band of phage T4 was seen only in Figure 2b, as expected. However, as MB concentration was increased above 2.5 mM, the band of phage T4 was progressively lost, in contrast to previous results [55] with bleomycin. At 5 mM MB, some lost phage band intensity was at the origin in Figure 2a, i.e., before the phage DNA was expelled for Figure 2b. This observation is explained by the MB permeabilization to GelStar of phages that were at the origin because of aggregation. The aggregates were apparently too large to enter the gel.

As the MB concentration was further increased, the origin-associated fluorescence was also lost, a further indication that the origin-associated DNA was in aggregated phages. Specifically, at the highest MB levels, loss of fluorescence at the origin was explained by the formation of aggregates too large to enter the agarose gel. Phage aggregation is confirmed below. Because of the possibility of aggregation, electron microscopy, rather than solution chemistry, was the optimal way to learn more about what the MB was doing to the phage T4.

### 2.2. Electron Microscopy (EM) by Negative Staining

EM confirmed the aggregation for the phage T4 that had been incubated with 10.0 mM MB and then negatively stained. Scanning of the specimen revealed that the dominant feature was the presence of aggregates. The aggregates were not present before incubation with methylene blue. These observations confirmed what was seen by AGE.

**Figure 3:**
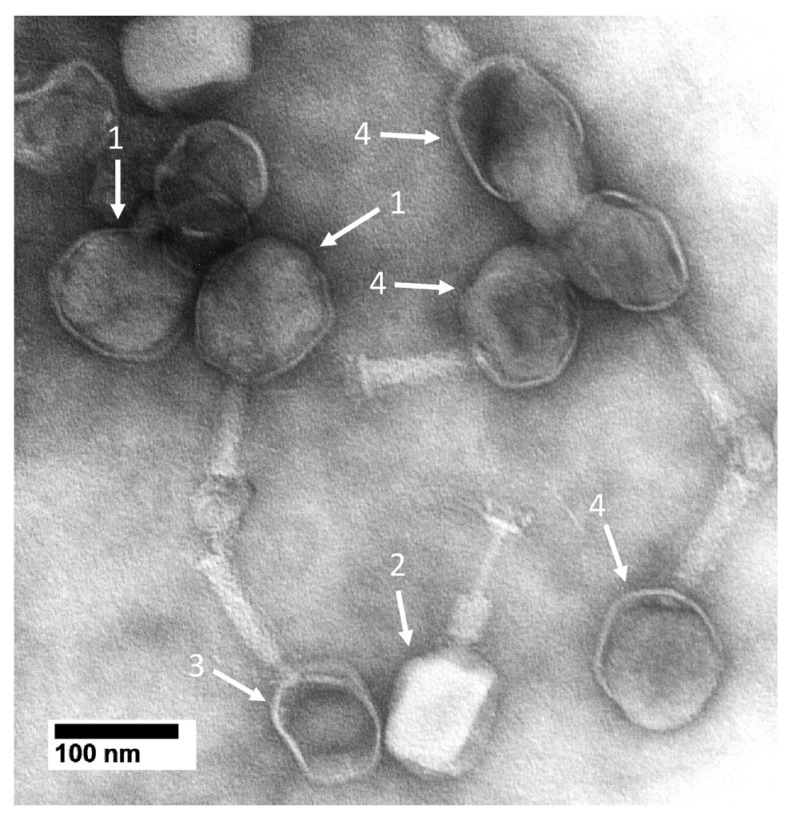
Electron microscopy of MB-reacted, negatively stained phage T4. Phage T4 was incubated with 10.0 mM MB, as done in Figure 2, and then immediately negatively stained with 1.5% uranyl acetate [55]. Images were obtained with a JEOL100CX electron microscope (JEOL USA, Inc., Peabody, MA, USA) operated at 80 kV.

The aggregates had primarily bacteriophages and phage heads. Some phages had atypical heads (e.g., #1 arrows in Figure 3). These heads appeared rounder and larger than the heads of a typical phage particle (arrow #2 in Figure 3), some of which had expelled the DNA genome (arrow #3 in Figure 3).

For the particles indicated by the #1 arrows in Figure 3, the exclusion of stain by packaged DNA was less than it was for particles with an apparently intact head (arrow #2 in Figure 3). The likely explanation is that the #1 arrow particles had a hyper-expanded outer shell, as confirmed by the increase in the size of the images of these particles. Particle flattening increases the apparent size but is not known to alter the apparent shape of negatively stained phages [57]. The increase in the size of #1 arrow particles in Figure 2a was great enough to favor the assumption of a real increase in size. This assumption is tested below.

Particles indicated by the #4 arrows in Figure 3 appeared to have started a hyper-expansion-related transition but not to have completed this transition. The result was that a bulge was present. This was confirmed by the decreased density of the DNA and the non-expulsion of the DNA. Both total and incomplete hyper-expansion required the thinning of a head’s outer protein shell. Thinning was suggested at the positions of the #1 arrows. However, the use of negative staining limited resolution to about 2 nm, which limited the reliability of this observation. Both shell thinning and shell size increase were further pursued by cryo-electron microscopy (cryo-EM), which did not cause either particle flattening or shape change.

### 2.3. Cryo-Electron Microscopy (Cryo-EM)

**Figure 4:**
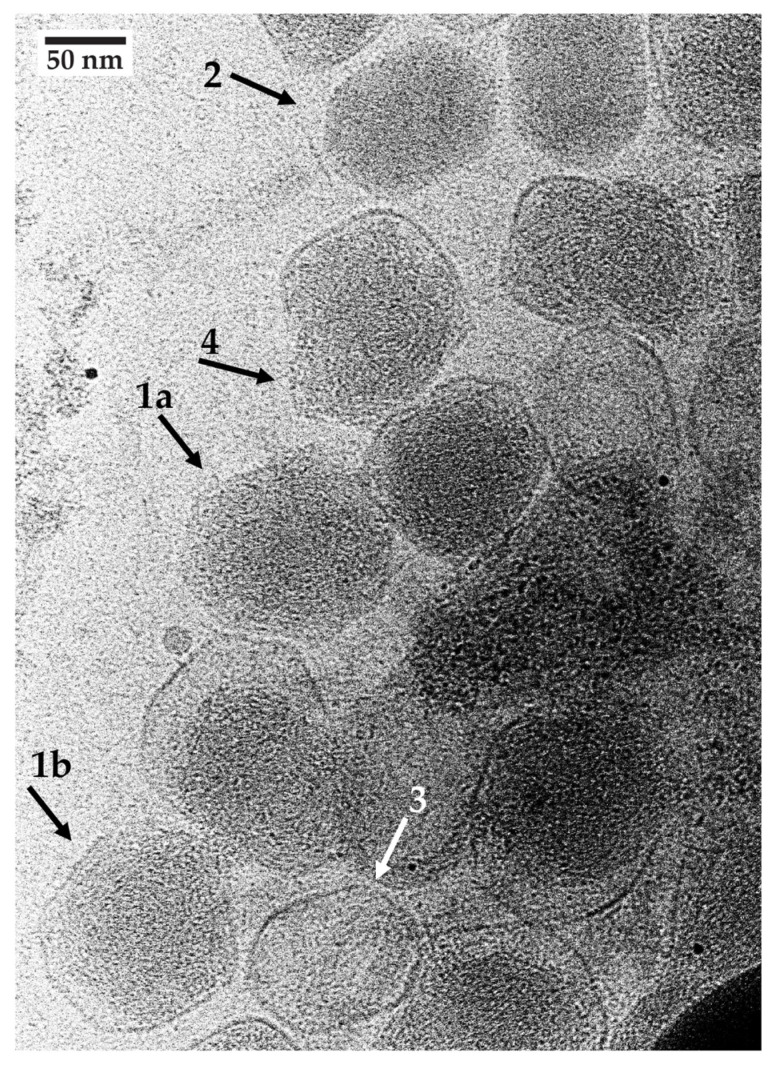
Cryo-electron microscopy of MB-reacted, unstained phage T4. Phage T4 was incubated with 10.0 mM MB as in Figure 2 and then immediately prepared for cryo-EM by use of the following procedure. The support was a Quantifoil copper holey carbon grid (R3.5/1, 200 mesh, Electron Microscopy Sciences, Hatfield, PA, USA) with a 2 nm ultrathin carbon layer. Grids were glow-discharged at 20 mA for 30 s in a Quorum EMS glow discharge machine (Iowa City, IA, USA). Then, a 3 ul phage sample was applied to a grid. The samples were blotted for 3 s with a blot force of 10 with filter paper under 100% humidity and a temperature of 8 °C in the Vitrobot Mark IV (Thermo Fisher Scientific, Waltham, MA, USA) chamber. Grids were plunge-frozen in liquid ethane prior to immediate transfer into liquid nitrogen for storage. Imaging was performed with a 200 kV Glacios cryo-TEM microscope (ThermoFisher Scientific, Waltham, MA, USA) with a gun lens of 4 and a spot size of 7. A 50 uM C2 aperture was used to adjust a parallel beam. All of the beam parameters were adjusted with a Falcon4 camera plus Selectris energy filter (ThermoFisher Scientific) by using Thermo Fisher Scientific EPU software, version 3.7. The beam parameters were the following: Atlas 110 X, Square 540 X, Hole 7600 X. The image acquisition magnification was 63,000 X with a pixel size of 1.89 Å, field of view of 775.4 nm and 10 eV slit width. After the grid Atlas map acquisition, a target-thinner ice square was chosen to map the whole picture, where target holes were used for the final data. Image acquisition was performed with MRC image file format. Images were recorded with an exposure time of 10 s, total dose of 20.22 e^−^/Å^2^ and dose rate of 2.02 e^−^/pix/s and the defocus range was manually adjusted from −2.5 to −2.0.

Cryo-EM confirmed the presence of (1) aggregates similar to those seen by negative staining, (2) separately observable particles at the edges of aggregates and (3) apparent reduced DNA density and hyper-expansion for some, but not all, particles. The hyper-expanded particles (e.g., arrow #1a,b in Figure 4) had a more isometric profile than the oblong, presumably unaltered phages, one of the latter indicated by arrow #2 in Figure 4. The DNA-containing shell diameter for the #1 arrow particles was 8–20% larger than the length, 115–120 nm, of the long axis of the phage heads, such as the one indicated by arrow #2 in Figure 4. The short axis of the arrow #2 shells was 84–88 nm. Thus, the arrow #1a,b particles were hyper-expanded, assuming that shell breakage was not the source of an apparent hyper-expansion.

Three observations indicated that many, if not all, of the #1 arrow particles did not have broken shells. First, no sign of breakage existed, and the outer shell was thinned to 1–2 nm (e.g., particle indicated by arrow #1a in Figure 4) in relation to the ~3 nm thick shells of capsids that had lost their DNA (arrow #3 in Figure 4). Shell thinning is a necessary consequence of hyper-expansion because of the conservation of mass. Second, when DNA appeared to have been expelled, the capsid appeared unbroken (arrow #3 in Figure 4). Third, some particles had a bulge where DNA appeared to be pushing against the capsid’s shell. However, the DNA appeared to still be restrained, as though the particle’s protein shell was still present to restrain the DNA (arrow #4 in Figure 4). If the shell had broken, the DNA would have been expelled.

Shells at a bulged region were expected to be thinned and more difficult to observe. Indeed, the bulge at arrow #4 in Figure 4 appeared to have a barely visible shell of about 1 nm in thickness at the tip of arrow #4. The opposing region of this shell (top of particle indicated by arrow #4) appeared to be thickened. The thickening can be explained by the binding of MB (positively charged [51]) to the shell, which, in turn, can be explained by the repeated negative charge on the shell as a consequence of the α-sheet structure. This explanation includes thinning of the opposing shell region via MB association and subsequent dissociation. Of course, more work is needed to rigorously determine the structure of the subunits of the thinned shells.

## 3. Where Next in Developing Strategies for AD? (Opinion)

### 3.1. Model and Implied Path to Improved Diagnosis

The above discussion supports the call to adjust the theoretical framework for developing therapies for both AD and other amyloid-associated neurodegenerative diseases. Specifically, the past strategy of removing or inactivating amyloid proteins is (1) not logical in the context of the above discussion, although it is the dominant current strategy, and (2) basically makes use of a theory that has been constructed without the use of data significantly beyond what Alzheimer [58] and Fischer [59] published over 100 years ago (review [60]). In more general terms, “It can scarcely be denied that the supreme goal of all theory is to make the irreducible basic elements as simple and as few as possible without having to surrender the adequate representation of a single datum of experience.” (Albert Einstein [61]). The latter part of this quotation is the key here.

Our proposed strategy for developing chemotherapy is based on an updated model of the source of toxicity and, therefore, the drug target (Figure 1). This model was constructed from data as comprehensive as we found possible by searching current publications. This model is, however, not proven. It has the advantage of having several aspects that can be tested.

First, the structure of subunits of the MB-altered, hyper-expanded, DNA-containing T4 shells should be determined. The primary current barrier to doing this is shell heterogeneity. Thus, one of our key objectives is obtaining unaggregated particles of this type with more uniform shells for 3-D reconstruction by cryo-electron microscopy. If the subunits are indeed found to have a version of the α-sheet structure, they will have the first α-sheet structure longer than 4–6 amino acids that has been seen by procedures other than molecular dynamics simulation [24,25,29,30].

Second, given the existence of appropriate human samples in brain banks, more extensive analysis should be performed of lipofuscin-associated and other neuronal fibers in the AD brain. The data cited in Section 1.1 constitute preliminary circumstantial evidence in favor of the idea that the key diagnostic criterion and key therapeutic target are α-sheets, not β-sheets. This past EM of the AD brain is suggestive but should be expanded.

Third, if MB is indeed found to be a probe for the toxic form of amyloid proteins, then work on MB–amyloid protein binding might converge with whole-body fluorescence imaging to produce a live patient assay for AD. MB does exhibit near-infrared fluorescence emission that is the basis of whole-body fluorescence imaging [62]. That was part of our original motivation for testing the loading of MB in phage T4.

Finally, in our opinion, the key remaining challenge for determining the pathway of AD disease is to track the disease process in vivo at its sub-clinical stages. This challenge includes developing tracking procedures that are sensitive to the dynamics of protein conformation. If such an in vivo assay were developed, it could also be used to determine the amount of α-sheet vs. the use of various therapies, i.e., to determine the effectiveness of a therapy. If MB was the therapeutic used, success would be indicated by a progressive decrease in the fluorescence signal from MB.

### 3.2. Implied Path to Improved Therapy

If MB affinity for T4 is an α-sheet affinity, the following would explain at least part of the observed AD-therapeutic effect of MB. This effect occurs via the blocking of the toxic activities of α-sheet-structured amyloid proteins. Evidence exists that these toxic activities include the formation of membrane channels [63]. However, if such binding stabilizes α-sheets, the model of Figure 1 indicates that this binding could aggravate the disease. Also, even if MB is always net therapeutic, MB might be far from the most potent molecule to use.

Thus, rapid initial screening for more effective, MB-related drugs is appropriate in our opinion. If MB-binding to T4 is specific to α-sheets, the procedure in Figure 2 can be adapted to screen for such compounds. Knowledge of T4 drug-binding vs. drug structure would be used to project the direction that drug improvement should take. Screening beyond this would also be added if other therapeutic effects, such as increasing mitochondrial oxidation by acting as a terminal electron receptor (already observed for MB [64]), are needed. Modifying MB and conducting the above screening would be based on the model of Figure 1 and, theoretically, is likely to be more successful than trying to eliminate amyloid-forming proteins.

Alternatively, MB-bound T4 phage particles might have therapeutic effects via the assembly of T4 α-sheets with excess amyloid α-sheets. To simplify the discussion below, protein assembly via an intrinsically self-assembling protein conformation, such as assembly to form either β-sheets or α-sheets, will be called conformation-derived assembly.

Therapeutic conformation-derived assembly is possibly embedded in the following observations of others. (1) A filamentous phage is found to have anti-amyloid protein assembly effects in murine models of Parkinson’s disease [65,66]. (2) A protein at the tip of the filament, gp3, is the active component [66]. This work was, however, performed in the context of immunotherapy, assuming the misfolding of amyloid proteins, and without considering either the potential conformation-derived assembly activity or the potential α-sheet structure of gp3 [67] (review [68]). The discussion above suggests the therapeutic activity of gp3 via α-sheet-driven, conformation-derived assembly with amyloid-forming proteins.

## 4. Relationship to the Development of Phage T4 as an Anti-Tumor Drug Delivery Vehicle

The following are the details for the use of phage T4 as a gated drug delivery vehicle. (1) The drug is loaded by opening the gate. (2) Transportation and inoculation are performed after closing the gate. (3) Drug release in a tumor is achieved by re-opening the gate. Means for (1) and (2) with phage T4 have been developed [55]. However, tumor-specific gate-opening is still a work in progress.

A possible strategy for in-tumor gate-opening is to co-load, in T4, MB that has been modified by attachment to a molecular group that blocks the activities of MB, including activity in capsid-permeabilization. The concept is to have the blocking group sensitive to removal specifically in the tumor environment. This environment includes relatively low pH, high lactate concentration, low pH-induced proteolytic activity (Warburg effect: reviews [69]) and high glutamine concentration (review [70]). If the chemistry for doing this were developed, it should be applicable no matter what drug is to be DDV-delivered.

## 5. Conclusions

As previously mentioned [68], work on biomedicine has the potential to introduce therapies that were not anticipated when the work started. The reasons for this are the complexity of biology and the associated limitation of our knowledge of how biology works. Thus, as we test models and therapies for non-infectious diseases, an open mind is critical for developing therapies. The work presented in Figure 2, Figure 3 and Figure 4 is an example.

## Data Availability

The data presented in this study are all available within one or more of the following: Figure and Text.
